# Human Islet Amyloid Polypeptide (hIAPP) Protofibril‐Specific Antibodies for Detection and Treatment of Type 2 Diabetes

**DOI:** 10.1002/advs.202202342

**Published:** 2022-10-18

**Authors:** Angelina S. Bortoletto, W. Vallen Graham, Gabriella Trout, Alessandra Bonito‐Oliva, Manija A. Kazmi, Jing Gong, Emily Weyburne, Brandy L. Houser, Thomas P. Sakmar, Ronald J. Parchem

**Affiliations:** ^1^ Center for Cell and Gene Therapy Stem Cells and Regenerative Medicine Center Department of Neuroscience Department of Molecular and Cellular Biology Translational Biology and Molecular Medicine Program Medical Scientist Training Program Baylor College of Medicine One Baylor Plaza Houston TX 77030 USA; ^2^ Laboratory of Chemical Biology & Signal Transduction The Rockefeller University 1230 York Avenue New York NY 10065 USA; ^3^ Celdara Medical 16 Cavendish Court Lebanon NH 03766 USA; ^4^ Department of Neurobiology, Care Sciences, and Society Center for Alzheimer Research Division of Neurogeriatrics Karolinska Institutet Solna 17164 Sweden

**Keywords:** amyloidosis, diabetes, monoclonal antibodies, therapeutics

## Abstract

Type 2 diabetes mellitus (T2D) is a major public health concern and is characterized by sustained hyperglycemia due to insulin resistance and destruction of insulin‐producing *β* cells. One pathological hallmark of T2D is the toxic accumulation of human islet amyloid polypeptide (hIAPP) aggregates. Monomeric hIAPP is a hormone normally co‐secreted with insulin. However, increased levels of hIAPP in prediabetic and diabetic patients can lead to the formation of hIAPP protofibrils, which are toxic to *β* cells. Current therapies fail to address hIAPP aggregation and current screening modalities do not detect it. Using a stabilizing capping protein, monoclonal antibodies (mAbs) can be developed against a previously nonisolatable form of hIAPP protofibrils, which are protofibril specific and do not engage monomeric hIAPP. Shown here are two candidate mAbs that can detect hIAPP protofibrils in serum and hIAPP deposits in pancreatic islets in a mouse model of rapidly progressing T2D. Treatment of diabetic mice with the mAbs delays disease progression and dramatically increases overall survival. These results demonstrate the potential for using novel hIAPP protofibril‐specific mAbs as a diagnostic screening tool for early detection of T2D, as well as therapeutically to preserve *β* cell function and target one of the underlying pathological mechanisms of T2D.

## Graphical abstract

Two monoclonal antibodies (mAbs) are reported that bind human islet amyloid polypeptide (hIAPP) protofibrils and inhibit their aggregation and toxicity to pancreatic islets in a mouse model of rapidly progressing type 2 diabetes mellitus (T2D). The mAbs detect protofibrils in diabetic animals and mAb treatment causes impressive survival increases. These data introduce the possibility of new diagnostic and therapeutic avenues in T2D.

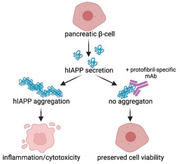

## 1 Introduction

Type 2 diabetes mellitus (T2D) is a metabolic disease characterized by a dysfunction in the body's ability to regulate blood glucose levels. If left untreated, high blood glucose, termed hyperglycemia, can lead to widespread complications affecting nearly every organ system in the body. T2D currently affects over 400 million people worldwide, and this number is estimated to increase to over 700 million by the year 2045.^[^ ^1^ ^]^ It is also predicted that the prevalence of impaired glucose tolerance, also referred to as prediabetes, will increase to over 500 million people globally. These individuals are characterized by significantly reduced insulin secretion in response to glucose stimulation.^[^ ^2^, ^3^ ^]^ Of these prediabetic individuals, at least 70% will progress to overt T2D in their lifetime.^[^ ^1^ ^]^

Although many questions remain regarding mechanism and etiology, one pathological hallmark of T2D seen in over 90% of patients is the toxic accumulation of human islet amyloid polypeptide (hIAPP) fibrils in pancreatic islets.^[^ ^4^ ^]^ Biologically active monomeric hIAPP is co‐secreted with insulin and functions as a hormone that aids in glucose uptake and satiety signaling.^[^ ^5^ ^]^ The receptors for hIAPP are heterodimeric complexes between the calcitonin receptor, a G‐protein‐coupled receptor, and one of three receptor activity‐modifying proteins (RAMPS), and are expressed mainly in the brain.^[^ ^6^, ^7^ ^]^ As the demand for insulin increases in diabetic and prediabetic patients, a simultaneous increase in hIAPP production and secretion occurs as well. hIAPP is one of the most aggregation‐prone peptides naturally occurring in the human body, and because of this, minimal increases in hIAPP levels can lead to the formation of hIAPP oligomers, which are toxic to insulin‐producing *β* cells.^[^ ^8^, ^9^, ^10^, ^11^, ^12^ ^]^ Although there is one known disease‐causing mutation in hIAPP, most diabetic individuals do not have any mutation in hIAPP. This suggests that any pathology associated with hIAPP is driven by its misfolding and aggregation, the structure and kinetics of which have been extensively reviewed.^[^ ^13^ ^]^ As T2D is a multifactorial disease, the toxic effects of hIAPP aggregation on *β* cells are not the only etiology of disease. However, hIAPP aggregation has clearly been shown to cause *β* cell dysfunction and death, and any potential mechanisms to prevent hIAPP aggregation are of great interest to the T2D field.^[^ ^14^ ^]^ Furthermore, any reduction in the number and functionality of *β* cells can exacerbate any underlying inadequacies in glucose control and causes a pathological cycle of increased hIAPP secretion and progressive *β* cell dysfunction seen in T2D.^[^ ^15^, ^16^, ^17^ ^]^

Current therapies for T2D do not address this key pathological feature, and in some cases, including the use of secretagogues that increase insulin secretion, hIAPP fibril deposition is exacerbated.^[^ ^4^ ^]^ At the early stages of hIAPP amyloidosis, soluble toxic protofibrils can escape the pancreas and circulate systemically; however, deposition of hIAPP protofibrils and formation of plaque‐like fibrils decrease diffusion of hIAPP out of the pancreas and simultaneously result in increased infiltration of pro‐inflammatory macrophages, yet reduced penetration of plaques by macrophages.^[^ ^18^, ^19^ ^]^ Ultimately, this culminates in a steep increase in amyloidosis progression. Although research has suggested that an immune‐mediated response to hIAPP protofibrils may serve as a promising therapeutic target,^[^ ^20^ ^]^ currently, there are no treatments that address amyloid aggregation in T2D.

One strategy to halt the toxic accumulation of hIAPP fibrils is the prophylactic approach of blocking their formation with monoclonal antibodies (mAbs). Blocking mAbs have been developed against protein mediators of hIAPP protofibril accumulation, such as the receptor for advanced glycation end products (RAGE) but have shown limited protection against disease progression.^[^ ^21^ ^]^ In addition, no mAbs have been developed specifically against hIAPP protofibrils as opposed to monomeric hIAPP. This approach has also proven difficult in other amyloid‐driven diseases in part due to the failure of previous mAbs to bind specifically to the oligomeric or protofibrillar forms of amyloids as opposed to their monomeric forms that function physiologically. Conformation‐specific mAbs against amyloid aggregates aim to overcome this barrier and have successfully been used to detect pathologic forms of amyloid in Alzheimer's disease.^[^ ^22^, ^23^, ^24^, ^25^ ^]^ Multiple mAbs have also shown promise in reducing amyloid burden in human samples.^[^ ^26^ ^]^ Aducanumab and solanezumab mAbs that bind the pathologic form of the Alzheimer's disease‐related amyloid, A*β*, are two such drugs that have shown promise in human clinical trials, with Aducanumab recently being approved by the United States Food and Drug Administration (FDA).^[^ ^27^ ^]^ However, discovering mAbs specific to the protofibrillar form of amyloid proteins is challenging and requires innovative approaches. Aducanumab was discovered by isolating mAbs from B‐cells harvested from cognitively normal elderly adults with subsequent screening for amyloid aggregate specificity.^[^ ^27^ ^]^ In addition, mAbs isolated from mice immunized for the *N*‐terminus of hIAPP have shown some prevention of islet fibril accumulation, lending strong support for an antibody‐mediated approach to protofibril aggregation prevention as a viable therapeutic approach.^[^ ^28^ ^]^ However, currently, there are no published mAbs known to be specific for early hIAPP protofibril species, as the protofibrillar form of hIAPP is transient and is difficult to isolate. Such mAbs may have potential use in diagnostic assays and in the development of novel therapies aimed at preventing or ameliorating disease progression in T2D.

We have developed a platform technology that stabilizes soluble amyloid protofibrils from varying amyloidogenic peptide sources using a modified chaperone‐like amyloid‐binding protein, nucleobindin 1 (NUCB1).^[^ ^29^ ^]^ We have previously used this technology to discover pan‐amyloid anti‐protofibril mAbs as well as amyloid‐specific anti‐protofibril mAbs, which were used to successfully detect A*β* protofibrils in a transgenic mouse model of AD as well as in human AD tissue.^[^ ^30^, ^31^ ^]^ Thus, we sought to determine if hIAPP‐specific anti‐protofibril mAbs could similarly be used for the detection of hIAPP protofibrils in T2D. However, in this study, we also aimed to test the potential for the diagnostic and therapeutic use of conformation‐specific mAbs in T2D. Here, we employ our protofibril‐stabilizing technology to create hIAPP protofibril immunogens for mouse immunization trials. Using the stabilized hIAPP protofibril immunogen, we raised mAbs in mice and selected clones with high affinity and specificity for hIAPP protofibrils. The mAbs were evaluated in vitro and two candidates were chosen for testing in a mouse model of T2D, in which a human transgene for hIAPP is overexpressed in mouse islet *β* cells. The two mAbs tested in the diabetic mice were able to detect hIAPP protofibrils in mouse serum and islets, confirming target engagement. Treatment of diabetic mice with either of the two mAbs also delayed disease progression and dramatically increased overall survival.

## 2 Results

### 2.1 Immunization with Stabilized hIAPP Protofibrils Produces Protofibril‐Specific mAbs

**Figure 1:**
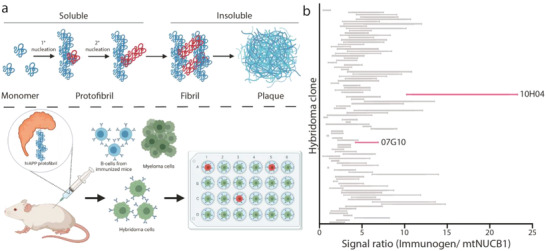
Purified mAb raised against *mt*NUCB1‐capped hIAPP bind capped hIAPP protofibrils in vitro. a) Top panel: hIAPP aggregation schematic showing the progression of hIAPP monomers to fibril and plaque formation (not drawn to scale). Bottom panel: *mt*NUCB1‐capped hIAPP protofibrils were used as the immunogen for mAb discovery. b) 104 supernatant fractions from successfully growing clonal hybridomas were analyzed for binding to either *m*tNUCB1‐capped hIAPP protofibrils or *mt*NUCB1 alone. Ratios of binding are shown. Pink‐labeled clones were selected for further analysis. Panel (a) was created with BioRender.

We have previously shown that a chaperone‐like amyloid‐binding protein, NUCB1, can be engineered to inhibit the aggregation of multiple sources of amyloid peptides through binding and stabilization of short protofibrils by a hypothesized capping mechanism.^[^ ^29^ ^]^ In this study, we used the engineered NUCB1 (*mt*NUCB1) to stabilize hIAPP protofibril complexes in solution, which were purified and used as the immunogen for the discovery of mAbs specific for hIAPP protofibrils (**Figure** **1**a). The immunogen was injected into BALB/c mice, which subsequently showed a robust titer to the *mt*NUCB1‐capped hIAPP protofibrils as well as *mt*NUCB1 alone (Figure S1, Supporting Information). B‐cells from immunized mice were isolated and clonal hybridoma cell lines were created.

To discriminate the relative binding of mAbs to hIAPP protofibrils and *mt*NUCB1, we screened supernatant fractions from the 104 best‐growing hybridoma clones using enzyme‐linked immunosorbent assay (ELISA) to measure the relative ratio of mAb binding of *mt*NUCB1‐capped hIAPP protofibril immunogen to *mt*NUCB1 alone (Figure 1b). Based on the mAb‐binding profiles, 40 mAbs were purified for subsequent analysis, and two were chosen as candidate mAbs for additional study.

### 2.2 Protofibril‐Specific mAbs Inhibit hIAPP Protofibril Aggregation

Amyloid aggregation can occur through multiple mechanisms including primary nucleation, fibril fragmentation and elongation, and secondary nucleation, which involves branching.^[^ ^32^, ^33^, ^34^ ^]^ Inhibition of these mechanisms results in changes in aggregation kinetics that can be measured using a Thioflavin T (ThT) fluorescence aggregation assay.^[^ ^35^ ^]^

**Figure 2:**
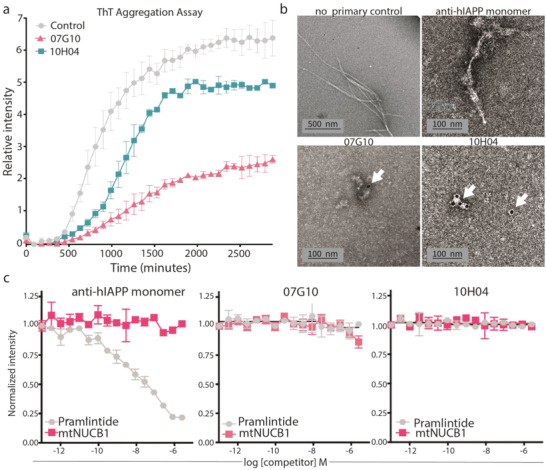
The mAbs inhibit aggregation and are specific for hIAPP protofibrils and not monomers. a) Kinetic analysis of ab inhibition of hIAPP aggregation via Thioflavin T (ThT) assay. The initial hIAPP monomer concentration for each experiment was 2.5 µm; ThT concentration was 10 µm; and each mAb was 1 µm. *n* = 2 per group. b) Immunogold electron microscopy analysis of no‐primary control, monomer control, 07G10, or 10H04 mAbs that were co‐incubated with hIAPP for 24 h to allow for aggregation. Candidate mAbs prevent fibril formation shown in the first part of panel (b). Only candidate mAbs co‐localize with protofibrils, as indicated by the electron‐dense gold particles (white arrows). c) Competition ELISA of mAbs to pramlintide or *mt*NUCB1 (half‐log dilution starting from 10 µm). Immunogen was coated at 2.5 µg mL^−1^ and anti‐hIAPP; 07G10 and 10H04 antibodies were added at 0.4 nm; *n* = 4 per group; and error bars represent S.E.M.

To study the mechanism of hIAPP aggregation, we chose 2 out of our 40 purified mAbs that displayed varying degrees of hIAPP aggregation inhibition over time (**Figure** **2**a; Figure S2, Supporting Information).

The mAb 07G10 decreased the end‐stage total fluorescence by more than half of the value of the control condition. The mAb 10H04 delayed the increase in fluorescence, and only partially decreased end‐stage fluorescence compared with the control. The delayed increase in fluorescence was also similar to that shown by the polyclonal antioligomer antibody A11 (Figure S4, Supporting Information). Since the specific mechanism of inhibition (e.g., inhibition of primary nucleation, fibril fragmentation and elongation, and/or secondary nucleation) results in specific changes in the Thioflavin T aggregation curve, we performed fitting of the aggregation curves using Amylofit 2.0^[^ ^36^ ^]^ (Figure S3 and Table S1, Supporting Information). The resulting trendlines suggest aggregation driven by a dominating multistep secondary nucleation process, further supported by previous studies showing similar trends in large‐scale hIAPP aggregation experiments.^[^ ^37^ ^]^

### 2.3 mAbs Are Specific for hIAPP Protofibrils and Not hIAPP Monomers

To visualize mAb–protofibril interactions, immunoelectron microscopy was performed. The hIAPP synthetic peptide was co‐incubated with candidate mAbs or the control Ab, allowed to aggregate for 24 h, stained with a gold‐labeled secondary antibody, and imaged. The control condition showed the formation of hIAPP fibril material, while co‐incubation with candidate mAbs resulted only in the formation of short protofibril species (Figure 2b). The 07G10 and 10H04 mAbs co‐localized with hIAPP protofibril material as indicated by the electron‐dense gold particle markers. These data indicate that 07G10 and 10H04 largely prevent the progression of soluble hIAPP protofibrils into nonsoluble hIAPP fibrils through direct binding to small hIAPP protofibril aggregates.

As noted above, *mt*NUCB1‐capped amyloid protofibrils can be used to discover conformation‐specific antiprotofibril mAbs.^[^ ^29^, ^30^, ^31^ ^]^ The conformation‐specific anti‐hIAPP protofibril mAbs were further screened against monomeric hIAPP. Since monomeric hIAPP is highly prone to aggregation, we screened our candidate mAbs against pramlintide, a nonaggregating hIAPP analog.^[^ ^38^ ^]^ Using a competition ELISA system, mAbs were evaluated for binding to *mt*NUCB1‐capped hIAPP protofibrils in the presence of varying concentrations of pramlintide. mAbs 07G10 and 10H04 displayed no significant binding to pramlintide as compared to the control anti‐hIAPP Ab, which binds a linear epitope in the peptide (Figure 2c). The mAbs were also evaluated for binding activity to *mt*NUCB1 because of the presence of the *mt*NUCB1 stabilizer in the mAb isolation system. The 07G10 and 10H04 mAbs showed no significant binding to *mt*NUCB1 in this competition ELISA (Figure 2c).

### 2.4 mAbs Detect hIAPP Protofibrils in Murine and Human T2D

**Figure 3:**
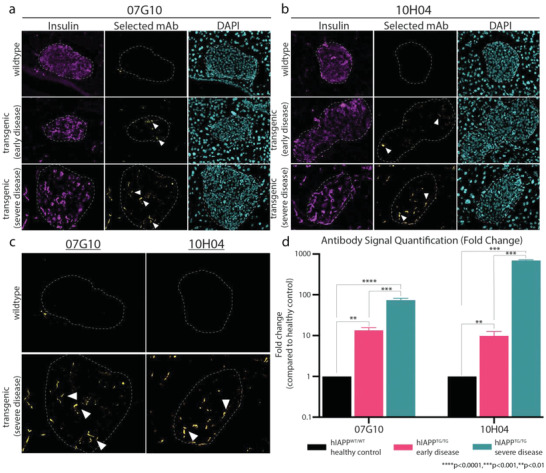
The mAbs engage hIAPP protofibrils in vivo. Immunofluorescence shows increased hIAPP protofibril staining with T2D disease progression with a) 07G10 and b) 10H04. Insulin (magenta), mAb (yellow), nuclei (cyan). c) Magnified image of 07G10 and 10H04 staining in WT and severe T2D. Protofibril staining is indicated by white arrowheads. d) Quantification of candidate mAb signal as the disease progresses compared to healthy controls. *n* = 3 per group; error bars represent S.E.M, ordinary one‐way ANOVA. ****p < 0.0001, ***p < 0.001, and **p < 0.01.

To determine the specific binding of selected mAbs to hIAPP protofibrils in vivo, we evaluated histological sections of pancreata from homozygous hIAPP transgenic mice (FVB/N‐Tg(Ins2‐IAPP)RHFSoel/J). This animal model expresses hIAPP under the regulatory control of the rat insulin II promoter^[^ ^39^ ^]^ and has been shown to spontaneously develop symptoms associated with T2D, such as *β* cell apoptosis, high blood glucose levels, insulin deficiency, and hIAPP fibril deposits.^[^ ^40^ ^]^ The T2D in transgenic mice begins to develop soon after weening and worsens as the mice age. After confirming clinical signs of diabetes and hIAPP expression (Figure S3, Supporting Information), we performed immunofluorescence to detect the presence of protofibrils using our candidate mAbs. The results indicate that 07G10 and 10H04 did not stain wild‐type (WT) islets. However, in the transgenic animal, distinct puncta were observed within or near the islets, which were detected by the presence of positive insulin staining (**Figure** **3**a–c). In addition, as the severity of the disease increased, the quantity of 07G10 and 10H04 positive foci increased despite significant decreases in insulin expression (Figure 3d). This result further supports the binding specificity of 07G10 and 10H04 for toxic hIAPP protofibrils, since monomeric hIAPP should decrease with decreased insulin production.

**Figure 4:**
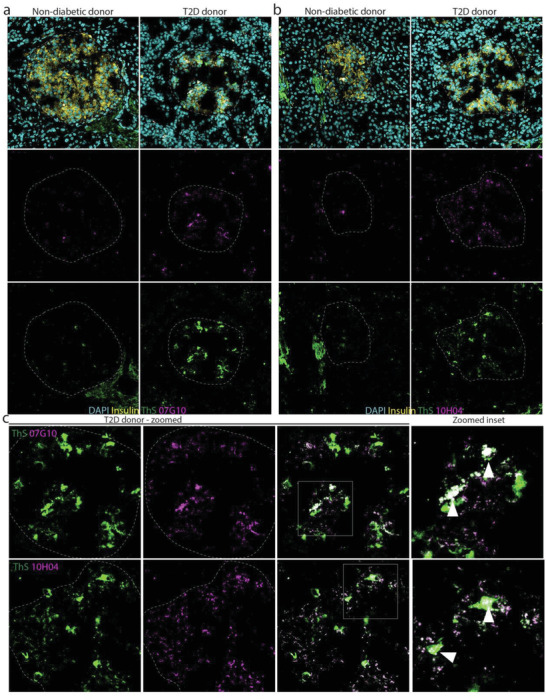
The mAbs engage hIAPP protofibrils in human T2D. Immunofluorescence shows increased hIAPP protofibril staining in pancreatic islet tissue from a T2D donor compared to nondiabetic control with a) 07G10 and b) 10H04. c) Magnified inset shows co‐localization of protofibril signal and hIAPP aggregate signal detected by ThS staining with protofibril signal localized at the core of larger aggregates. mAb (magenta), ThS (yellow), nuclei (cyan). Co‐localization of protofibril signal and ThS signal is indicated by white arrowheads.

To confirm that our mAbs can also detect protofibrils in human T2D, we obtained pancreatic sections from healthy and T2D donors (Novus, Biochain) and performed immunofluorescence with our mAbs and Thioflavin S (ThS) staining to mark hIAPP aggregates. Some baseline level of signal with healthy donors is expected as tissue was acquired from donors of similar age. Additionally, even healthy individuals express hIAPP, so some level of protofibril signal is likely to occur, especially in donors of advanced age, such as in our case (Table S2, Supporting Information). However, compared to healthy donors, tissue from T2D donors showed increased ThS and anti‐protofibril mAb staining (**Figure** **4**a,b). ThS signal also overlapped with anti‐protofibril mAb staining (Figure 4c) and interestingly, the anti‐protofibril signal seemed to be at the core of larger aggregates (Figure 4c, inset, white arrows). The presence of protofibrils at the center of larger aggregates provides further support for our hypothesis that protofibrils promote either primary or secondary nucleation events, both of which can lead to the formation of larger hIAPP aggregates within pancreatic islets. This is also supported by the aggregation curves fitting trendlines suggestive of a multistep secondary nucleation‐dominated process.

**Figure 5:**
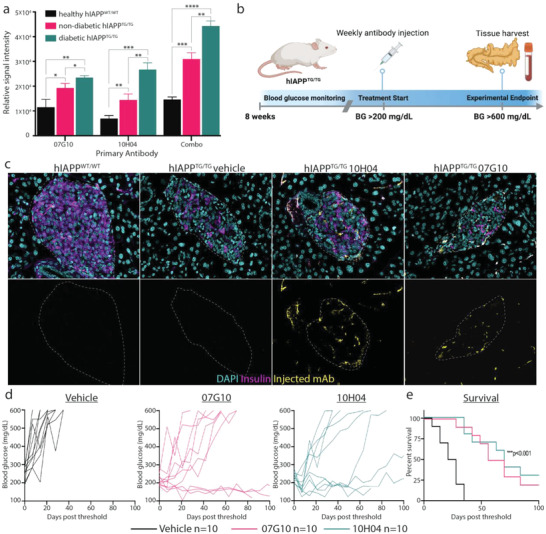
The mAbs detect hIAPP protofibrils in serum, delay disease progression, and increase survival. a) Treatment study experimental design. b) ELISA of mAbs in the detection of hIAPP protofibrils in serum from hIAPP^TG/TG^ mice. Serum from healthy WT controls only showed a background level of signal and the signal is increased in sick versus healthy transgenic mice. *n* = 3 per group. c) Immunofluorescence imaging of WT, vehicle‐treated, and Ab‐treated mice showing that mAbs can engage hIAPP protofibrils at pancreatic islets. Insulin (magenta), mAb (yellow), nuclei (cyan). d) Fasting blood glucose level in vehicle‐ and mAb‐treated mice (10 mg kg^−1^). Vehicle (black), 07G10 (magenta), and 10H04 (teal). e) Kaplan–Meyer survival analysis. *n* = 10 per group; error bars represent S.E.M., ordinary one‐way ANOVA or log‐rank test. ****p < 0.0001, ***p < 0.001, **p < 0.01. BG = blood glucose. Panel (a) was created with BioRender.

Next, to determine if the mAbs can detect circulating levels of hIAPP protofibrils in serum, we performed ELISAs with serum isolated from healthy (young, nondiabetic) hIAPP^TG/TG^ mice, diabetic hIAPP^TG/TG^ mice, and wild‐type hIAPP^WT/WT^ mice. The 07G10 and 10H04 mAbs showed minimal background signal in hIAPP^WT/WT^ mice. However, 07G10 and 10H04 showed increased binding to serum from prediabetic hIAPP^TG/TG^ mice and an even greater signal with serum from diabetic hIAPP^TG/TG^ mice (**Figure** **5**a), suggesting that mAbs can bind to circulating, soluble hIAPP protofibrils in serum. Because there is an increase in signal in diabetic serum samples compared with serum samples from young, nondiabetic mice, this suggests that our candidate mAbs may also be used to screen for prediabetes and as a marker of disease progression.

### 2.5 mAbs Prevent Disease Progression and Increase Survival in Murine T2D

Fasting blood glucose levels increase in the hIAPP^TG/TG^ mice, and increase as they age. First, to determine if mAbs can localize to and engage hIAPP protofibrils in pancreatic islets, we injected 10 mg kg^−1^ of 07G10 and 10H04 into the tail vein of two hIAPP^TG/TG^ mice. One week later we harvested pancreata and performed immunofluorescence to determine if we could detect mAbs in pancreatic islets (Figure 5b). Indeed, we were able to detect the presence of both mAbs in treated mice, but not hIAPP^WT/WT^ or vehicle‐treated hIAPP^TG/TG^ mice (Figure 5c). This result suggests that the candidate mAbs can engage hIAPP protofibrils at pancreatic islets after systemic injection and that this engagement is sustained over time, as they are still detectable 1 week after injection.

**Figure 6:**
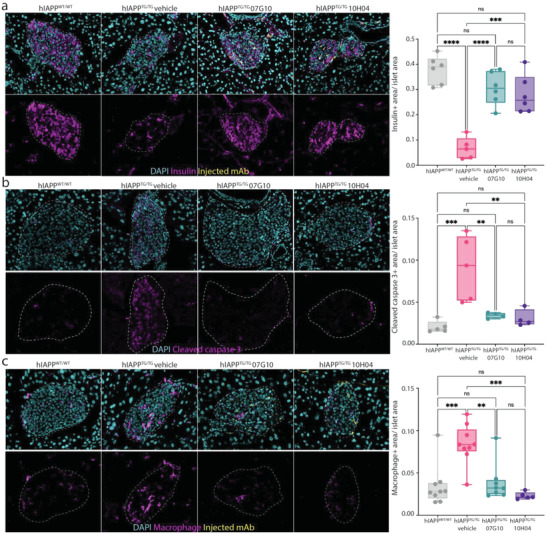
The mAbs protect islets from macrophage‐driven *β* cell destruction. a) Immunofluorescence imaging of pancreatic islets from WT, vehicle‐control, and mAb‐treated mice showing that mAb treatment preserved the islet health of treated mice. Insulin (magenta), mAb (yellow), nuclei (cyan). b) Immunofluorescence imaging of pancreatic islets from WT, vehicle‐control, and mAb‐treated mice showing mAb treatment prevented islet apoptosis cleaved caspase 3 (magenta), mAb (yellow), and nuclei (cyan). c) Immunofluorescence imaging of pancreatic islets from WT, vehicle‐control, and mAb‐treated mice showing mAb treatment prevented increased macrophage infiltration seen with diabetic mice. Macrophage (magenta), mAb (yellow), nuclei (cyan). *n* = 2 per group, technical triplicates; error bars represent S.E.M.; ordinary one‐way ANOVA. ****p < 0.0001, ***p < 0.001, and **p < 0.01, ns = not significant.

Next, we wanted to assess the therapeutic potential of the mAbs. Once fasting glucose levels in the hIAPP^TG/TG^ mice were >200 mg dL^−1^, we began weekly intravenous injections of each of the mAbs and continued to monitor weekly blood glucose levels until they reached >600 mg dL^−1^, at which time the mice were euthanized for tissue harvest. We observed a significant delay in disease progression (measured by fasting blood glucose levels) as early as 1 week after treatment with the candidate mAbs (Figure 5d). More importantly, we observed that mAb‐treated mice survived twice as long as vehicle‐treated mice (Figure 5e). To confirm that the increase in survival was due to the preservation of islet health, we examined pancreatic islets from mice with a robust response to antibody treatment. Pancreatic insulin expression of these mice was comparable to wild‐type mice, and islets appeared much healthier than vehicle‐treated mice which had severe diabetes (**Figure** **6**a). Additionally, in two robust responders, treatment was withdrawn after 10 weeks of treatment to determine how long treatment protected against disease. In the mouse treated with mAb 07G10, fasting blood glucose levels began to rise after ≈5 weeks without treatment, while mice treated with mAb 10H04 sustained a response until nearly 10 weeks after treatment withdrawal (Figure S6, Supporting Information). This result suggests that strong responders maintain protection against disease over multiple weeks, which may allow for fewer doses in future experiments. Together, these data suggest that candidate mAbs target hIAPP protofibrils specifically at pancreatic islets and in doing so prevent *β*‐cell toxicity caused by protofibril accumulation.

### 2.6 mAbs Preserve Islet *β* Cells and Prevent Increased Pancreatic Islet Macrophage Infiltration

Pro‐inflammatory macrophage infiltration has been shown to contribute to hIAPP‐protofibril‐mediated pancreatic islet damage.^[^ ^19^ ^]^ To confirm that mAb treatment prevented islet cell apoptosis, we investigated islet cleaved caspase 3 signal, which is a marker of programmed cell death. Cleaved caspase 3 expression was significantly increased in vehicle‐treated transgenic mice, by over fourfolds (Figure 6b). Next, to determine how treatment with mAb is protecting pancreatic islets from destruction, we looked at islet macrophage infiltration for one of the candidate mAbs (07G10). Macrophage infiltration was markedly increased in diabetic pancreatic islets from hIAPP^TG/TG^ mice (Figure S7, Supporting Information). However, in mice treated with mAb, increased macrophage infiltration was prevented, and macrophage infiltration levels in pancreatic islets were indistinguishable from wild‐type pancreatic islets (Figure 6c). In addition, we assessed if the mAbs could protect against hIAPP ‐mediated cytotoxicity independent of any inflammatory‐mediated effect by performing an in vitro cell viability assay. Cell viability of INS‐1 cells was assessed 24 h after the addition of the hIAPP peptide. Compared to control, both the addition of 07G10 and 10H04 led to a modest, yet significant increase in viability (Figure S8, Supporting Information). Together, these data suggest that the candidate mAbs prevent protofibril aggregation, while also preventing *β*‐cell damage from both the cytotoxic‐ and macrophage‐mediated inflammatory effects of hIAPP aggregation.

## 3 Discussion

Diagnostic and therapeutic application of hIAPP protofibril‐specific mAb targeting the pathological forms of soluble amyloid intermediates is a compelling approach to disease alleviation in T2D. To date, however, the identification and development of mAb specific for the extremely transient amyloid protofibrils have been a challenge. The present study describes the use of a novel platform^[^ ^29^ ^]^ in which *mt*NUCB1‐capped and stabilized hIAPP protofibrils are purified for use as the immunogen for the discovery of anti‐hIAPP protofibril mAbs. After immunization and clonal hybridoma production, supernatant fractions were used to identify the top‐performing cell lines. A total of 40 mAbs were purified for analysis, and two candidate mAbs, 07G10 and 10H04, were chosen to represent the two different and distinct profiles seen in kinetic aggregation experiments. Proteins that bind to different transient intermediates in the amyloid aggregation pathway can affect biochemical rate constants of aggregation kinetic reflected by changes in the conversion of monomer to fibril as measured over time by fibril‐sensitive dyes, such as Thioflavin T.^[^ ^35^ ^]^ Interestingly, differences in kinetic aggregation experiments between the mAbs did not translate to obvious differences in the in vivo assays carried out. This observation may be due to varying specificities of each mAb to different protofibril conformations, which may have different effects on the progression from the soluble protofibril forms of hIAPP aggregates. However, the in vivo assay may also simply be insensitive to the effects of mAb binding to various hIAPP protofibril intermediates, as long the overall aggregation process is disrupted, or if the hIAPP protofibrils are rendered less toxic when they are complexed with the mAbs.

A competition ELISA was used to verify that mAbs 07G10 and 10H04 did not demonstrate affinity to the stable monomeric form of hIAPP, pramlintide, or the “capping” protein, *mt*NUCB1. Immunogold electron microscopy (EM) analysis showed that each mAb bound a small amyloid aggregate of typical protofibril‐like size and shape. Using a transgenic mouse model of hIAPP‐induced diabetes, we observed that mAbs 07G10 and 10H04 detect aggregates near or in pancreatic islets, as well as in serum samples from the same mice. Additionally, mAbs 07G10 and 10H04 detected aggregates in human T2D tissue. Interestingly, when co‐stained with Thioflavin S, a fibril‐sensitive dye similar to Thioflavin T historically used in histological analysis; protofibrils seemed to primarily be located at the center of larger aggregates. This suggests that protofibril formation is important for the formation of larger aggregates. These data indicate that this model of hIAPP overexpression in *β* cells is associated with hIAPP aggregation and thus leads to islet dysfunction and an overt T2D disease phenotype.

The mAbs that target hIAPP protofibrils may prove to be useful for the diagnosis of hIAPP‐induced islet damage, or as an early screening modality for prediabetes. According to recommendations by the American Diabetes Association, screening for T2D and prediabetes should begin at age 45 for all asymptomatic adults and earlier for those who are overweight and have one or more risk factors.^[^ ^41^ ^]^ However, current screening modalities are relatively insensitive, as well as relatively intensive, and require fasting and multiple blood draws. More importantly, all current approaches for screening and disease monitoring depend on the presence of already existing pancreatic dysfunction and thus are monitoring pathology associated with T2D instead of early predisease biomarkers. We observed that the mAbs tested were able to detect hIAPP protofibrils in serum from hIAPP^TG/TG^ mice before the onset of clinical pathology. This suggests the potential to use the mAbs as a novel screening approach that could detect the earliest events preceding overt pancreatic dysfunction. An Ab‐based approach for early disease detection is also likely far more sensitive than current detection methods as we have shown the ability to detect early protofibril accumulation before any pancreatic dysfunction is present, at least as judged by serial fasting glucose monitoring. In addition, Ab‐based approaches do not depend on the administration of glucose or insulin, mitigating any variability due to the introduction of exogenous substances. Early detection of serum hIAPP protofibrils as a diagnostic biomarker may provide the opportunity for lifestyle modifications that could lead to the prevention of T2D onset or halt the transformation of prediabetes to T2D.

Finally, the discovery of hIAPP protofibril‐specific mAbs also has potential therapeutic implications. Treatment with anti‐hIAPP protofibril mAbs in the T2D mouse model not only decreased disease progression but doubled overall survival time. Translating these results into patients could provide an opportunity for systemic clearance of early‐onset, soluble aggregates of hIAPP, representing the first treatment that targets underlying pathology in T2D. Prevention of pathological amyloid aggregates may also reduce macrophage‐mediated islet inflammation, leading to the preservation of healthy pancreatic islet *β* cells.

## 4 Conclusion

We have described the discovery of conformation‐specific anti‐hIAPP protofibril mAbs using a novel platform that stabilizes soluble *mt*NUCB1‐capped hIAPP protofibrils for use as an immunogen. The resulting mAbs display varying effects on the modes of microscopic aggregation kinetics through binding protofibrils, but not monomeric epitopes. In addition, these mAbs are useful in detecting hIAPP aggregates ex vivo and can localize to pancreatic islets in vivo. Most excitingly, the mAbs allow for the detection of soluble, early hIAPP protofibril species in serum samples and delay disease progression and increase survival in a mouse model of T2D. These mAbs will be a useful tool for the study of hIAPP‐dependent islet dysfunction, and also represent an exciting opportunity for a new diagnostic tool for detecting early stages of the disease before irreparable damage has occurred, as well as a potential treatment that targets underlying pathology in T2D.

## 5 Experimental Section

### Preparation of hIAPP Peptide

The hIAPP synthetic peptide (Tables S3 and S4, Supporting Information) (Bachem) was solubilized in hexafluoroisopropanol (HFIP) at 1 µg µL^−1^ for 1 h at room temperature (RT) with vortexing every 10 min and sonicated in a water bath sonicator for 10 min. The peptide was aliquoted in low‐retention tubes (Fisher) at 30 µg per tube, dried with a speed vac, and stored at −80 °C.

### mtNUCB1‐Capped hIAPP Protofibrils

Recombinant expression of the engineered, soluble, and Ca^2+^‐free *s*NUCB1 (*mt*NUCB1) was previously described.^[^ ^30^ ^]^ 30 µm hIAPP was co‐incubated together with 10 µm *mt*NUCB1 in 20 mm sodium phosphate, pH 7.6, for 24 h, at 37 °C in quiescent conditions. The capped‐protofibril‐containing solution was then applied to a Superdex200 26/60 PG size exclusion chromatography (SEC) column (GE Healthcare) and equilibrated with 20 mm sodium phosphate, pH 7.6, 150 mm NaCl. The relevant peak was collected for subsequent experiments.

### Immunization Campaign

Three BALB/c mice were immunized subcutaneously and intraperitoneally with a total of 20 µg of freshly prepared *mt*NUCB1‐capped hIAPP protofibrils and boosted every 14 days for three additional times. Blood was collected 7 days after the second boost. Mice with high serum reactivity to *mt*NUCB1‐hIAPP were boosted one final time, 2 days after the third boost. Five days later, the mice were euthanized by low‐flow carbon dioxide overexposure followed by exsanguination by cardiac puncture, and spleens were harvested for hybridoma fusions.

### Hybridoma Fusions and Screening

The mouse spleens were fused with P3 myeloma cells (ATCC Cat # CRL1580) according to published methods.^[^ ^42^ ^]^ Hybridoma supernatants were screened for *mt*NUCB1 and *mt*NUCB1‐capped hIAPP protofibril reactivity using ELISAs. Of 104 supernatants screened, 40 hIAPP protofibril‐specific hybridomas were selected for further experimentation.

### Immunogold Electron Microscopy

The hIAPP peptide was diluted to 5 µm in the presence or absence of 5 µm mAb and incubated for 24 h, at 37 °C. A sample of 5 µL was placed onto a carbon film 200 mesh copper grid for 2 min, followed by a 3 min incubation with 3% bovine serum albumin (BSA). The grid was then incubated for 20 min with an antimouse 12 nm gold‐conjugated secondary antibody (Jackson Laboratories, 1:20). The grid was then extensively rinsed in buffer (20 mm sodium phosphate) and counterstained with a 2% aqueous uranyl acetate solution. Samples were viewed with a JEOL 1400 Plus transmission electron microscope (TEM) and images were acquired with Gatan 2K x 2K digital camera.

### Thioflavin T Binding Assay

The kinetics of aggregation of hIAPP (2.5 µm) was tested in the presence of 1 µm of the whole immunoglobulin G (IgG) mAb and 10 µm Thioflavin T (Fisher Scientific). A volume of 50 µL per well (*n* = 4 per group) was added to each well of a prechilled (4 °C) Corning 96‐well half area black with clear flat‐bottom polystyrene with a nonbinding surface (NBS) and covered with a clear self‐adhesive top seal. The aggregation was tested every 10 min for up to 48 h in quiescent conditions and at a constant temperature of 37 °C. Fluorescence measurements were performed on a Flexstation II (Molecular Devices) using an excitation wavelength of 450 nm and an emission wavelength of 485 nm. The obtained fluorescence measures were normalized to the relative fluorescence expressed after 20 min of incubation.

### ELISA

To measure the solution competition of antibodies to immobilized *mt*NUCB1‐capped hIAPP protofibrils by soluble monomeric hIAPP (pramlintide) or *mt*NUCB1, black 384‐well maxisorp plates (NUNC) were coated with immunogen diluted in coating buffer at a concentration of 2.5 µg mL^−1^ overnight at 4 °C. The following day the plate was washed and blocked with 1× Tris‐buffered saline (TBST, 0.1% Tween 20 detergent) containing 1% BSA for 1 h at RT and successively incubated with a fixed concentration (0.4 nm) of antibody together with decreasing concentrations of pramlintide monomers or *mt*NUCB1, starting at 10 µm and diluted in half‐logs. The binding was detected with appropriate horseradish peroxidase (HRP)‐conjugated secondary antibodies and Amplex UltraRed (Thermo).

For serum ELISAs, black 96‐well maxisorp plates (NUNC) were coated with serum samples diluted 1:50 in coating buffer overnight at 4 °C. The following day the plate was washed 3× with 1× phosphate‐buffered saline (PBS), 0.1% Tween 20 (PBST), and blocked with PBST containing 1% BSA for 1 h at RT. The plate was then incubated with primary mAbs (07G10, 10H04, or both combined) at a concentration of 2.5 µg mL^−1^ of each antibody for 2 h at room temperature. The plate was then washed 3× with PBST and incubated with the appropriate HRP‐conjugated secondary antibodies. The plate was again washed 3× with PBST and incubated for 30 min with Amplex UltraRed (Thermo) solution and read in a microplate reader.

### Blood Glucose Monitoring

Blood glucose concentrations were examined after a 14 h fast every 7 days. Values were measured from a tail‐tip blood sample by a Contour Next blood glucose meter (Ascencia Diabetes Care).

### Immunohistochemistry in Mouse Tissue

FVB/N‐Tg (Ins2‐IAPP) RHFSoel/J mice displayed hIAPP deposits in the pancreatic islets, resulting in a rapid onset of a diabetes phenotype.^[^ ^39^ ^]^ Adult (male) FVB/N‐Tg (Ins2‐IAPP) RHFSoel/J transgenic mice or wild‐type mice (FVBwt) were euthanized and pancreata were excised, fixed in 4% formaldehyde for 1 h at RT, and embedded in optimum cutting temperature compound (O.C.T.) (Sakura Finetek). Pancreata were then sectioned at 10 µm thickness and slides were stored at −80 °C until staining.

For immunohistochemistry, slides were warmed to RT, and then washed three times with 0.1% Triton‐X in PBS (PT) for 5 min each. Blocking (5% goat serum, 1% BSA in PBS) was done for 1 h at RT. The mAbs (07G10 and 10H04) were centrifuged at 15 000 × *g* for 5 min at 4 °C before being diluted to a concentration of 10 µg mL^−1^. Slides were co‐stained with either guinea pig anti‐insulin (Fisher PA126938, 1:100), mouse IgG1 anti‐insulin (Santa Cruz sc‐8033, 1:500), mouse IgG2a anti‐amylin (Santa Cruz sc‐377530, 1:500), or rat anti‐F4/80 (Abcam ab6640, 1:100) and were incubated overnight at 4 °C. After incubation, slides were washed five times with PT for 5 min, and then incubated for 2 h at RT with fluorescently conjugated secondary antibodies, Alexa Fluor goat anti‐guinea pig 647, Alexa Fluor goat antirat 594, and Alexa Fluor goat antimouse IgG2a 488 (Fisher). Slides were washed five times with PT for 5 min and incubated in 4',6‐diamidino‐2‐phenylindole (DAPI) (1 µg mL^−1^, Sigma) for 10 min. After three 5 min washes with PT, slides were mounted and images were visualized on an inverted fluorescent microscope (LSM 990 confocal microscope; Zeiss, Thornwood, NY, USA). Animal tissue sample collection and injection were approved by the Baylor College of Medicine Institutional Animal Care and Use Committee (AN‐8205) and performed in accordance with regulations and established guidelines.

### Immunohistochemistry in Human Tissue

Fresh frozen human pancreatic sections were obtained from Novus Biologicals (Centennial, CO) and BioChain (Newark, CA) and stored at −80 °C until staining. According to the manufacturer, tissues were snap‐frozen in liquid nitrogen immediately after excision and embedded in O.C.T. Tissues were then sectioned at 5–10 µm thickness and mounted on positively charged glass slides.

For immunohistochemistry, slides were warmed to RT, and then washed three times with 0.1% Triton‐X in PBS (PT) for 5 min each. For Thioflavin S staining (Millipore Sigma T1892‐25G, 0.1% in 50% ethanol), slides were washed once with 50% ethanol for 5 min then stained with Thioflavin S for 10 min. Slides were then washed three times for 1 min each with PT and then blocking (5% goat serum, 1% BSA in PBS) was done for 1 h at RT. The mAbs (07G10 and 10H04) were centrifuged at 15 000 × *g* for 5 min at 4 °C before being diluted to a concentration of 10 µg mL^−1^. Slides were incubated overnight at 4 °C with candidate mAbs and guinea pig anti‐insulin (1:100). After incubation, slides were washed five times with PT for 5 min, then incubated for 2 h at RT with fluorescently conjugated secondary antibodies Alexa Fluor goat antimouse IgG 546 (Fisher) and Alexa fluor goat antiguinea pig 647. Slides were washed five times with PT for 5 min and incubated in DAPI (1 µg mL^−1^, Sigma) for 10 min. After three 5 min washes with PT, slides were mounted and images were visualized on an inverted fluorescent microscope (LSM 990 confocal microscope; Zeiss, Thornwood, NY, USA). Animal tissue sample collection and injection were approved by the Baylor College of Medicine Institutional Animal Care and Use Committee (AN‐8205) and performed in accordance with regulations and established guidelines.

### Antibody Treatment of Mice

Weekly fasting blood glucose monitoring was begun on FVB/N‐Tg (Ins2‐IAPP) RHFSoel/J mice at 8 weeks of age. Once fasting blood glucose levels reached >200 mg dL^−1^, mice were randomly assigned to either vehicle‐, 07G10‐ or 10H04‐treated groups. Abs were injected once a week into the tail vein at 10 mg kg^−1^ in 200 µL or less of sterile physiologic grade saline or an equal volume of saline in vehicle‐treated mice. Weekly fasting blood glucose monitoring was continued until levels reached >600 mg dL^−1^, at which time mice were euthanized for tissue harvest.

### PrestoBlue Cell Viability Assay

INS‐1 cells (10 000 cells per well) were preincubated overnight in 96‐well plates. The cells were treated with 5 µm hIAPP in the presence or absence of mAbs 07G10 or 10H04 for 24 h. PrestoBlue cell viability assays (ThermoFisher) were performed by treating INS‐1 cells with PrestoBlue labelling solution for 30 min at 37 °C. Conversion of resazurin to resorufin was detected by quantifying fluorescence at 560/590 nm with a Victor plate reader (Perkin Elmer).

### Statistical Analyses

All experimental data were analyzed with GraphPad Prism and expressed as mean ± standard error of the mean (SEM). Differences among three or more groups were evaluated with a one‐way or two‐way analysis of variance (ANOVA) and Bonferroni post‐tests. Kaplan–Meyer survival analysis was evaluated with a log‐rank test for trends. *p* < 0.05 was considered significant.

## Conflict of Interest

T.P.S. and W.V.G. are listed as inventors on a US patent related to this study. The authors have no additional financial interests.

## Authors Contribution

A.S.B., R.J.P., W.V.G., and T.P.S. designed the study. W.V.G. and A.B.‐O. prepared and characterized the immunogens and conducted in vitro experiments with the purified antibodies along with M.A.K. and E.W. A.S.B. performed ex vivo immunofluorescence, serum ELISA experiments, antibody treatments, and macrophage staining experiments. G.T. contributed to serum ELISA experiments and immunofluorescence experiments. T.P.S., R.J.P., J.G., and B.L.H. supervised various aspects of the work. A.S.B., W.V.G., and A.B.‐O. wrote the paper with critical input from R.J.P. and T.P.S., and all other authors who approved the final version.

## Supporting information

Supporting Information

## Data Availability

The data that support the findings of this study are available from the corresponding author upon reasonable request.
