# Supplementary material for: Human Islet Amyloid Polypeptide (hIAPP) Protofibril‐Specific Antibodies for Detection and Treatment of Type 2 Diabetes
Source: Adv Sci (Weinh). 2022 Oct 18;9(34):2202342. doi: 10.1002/advs.202202342 (PMC9731688; doi:10.1002/advs.202202342)

Supporting Information

Human Islet Amyloid Polypeptide (hIAPP) Protofibril-Specific Antibodies for Detection and Treatment of Type 2 Diabetes

Angelina S. Bortoletto, W Vallen Graham, Gabriella Trout, Alessandra Bonito-Oliva, Manija A. Kazmi, Jing Gong, Emily Weyburne, Brandy L. Houser, Thomas P. Sakmar, Ronald J. Parchem*

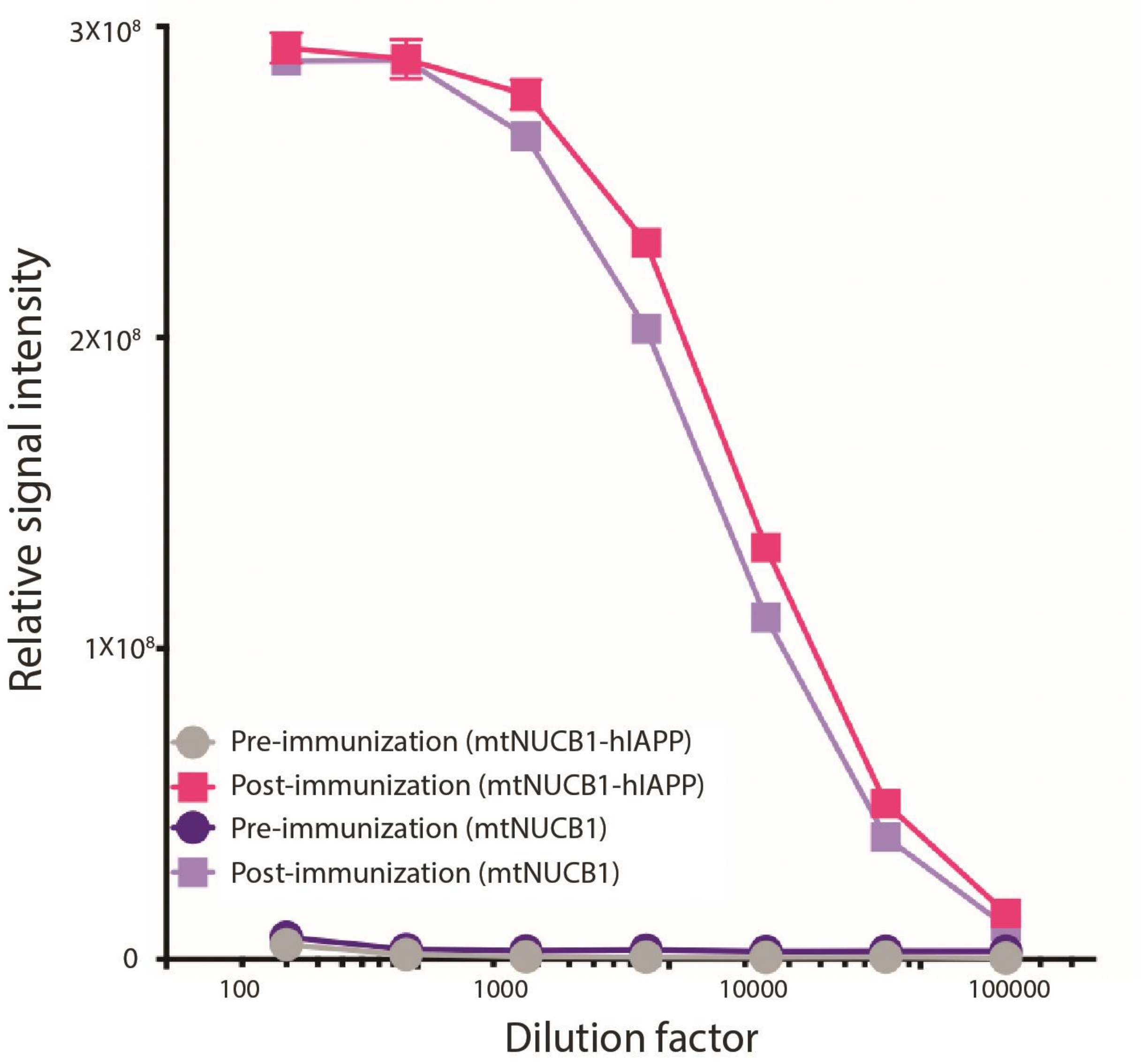

Figure S1. Immunization titer. *Mice were immunized with mtNUCB1-capped hIAPP protofibrils. Pre-immunization and post-immunization serum samples were collected to determine antibody titer to either the immunogen or the mtNUCB1 capping protein. One example titer is shown here. Serum levels are shown for pre-immunization antibodies against mtNUCB1-hIAPP (gray), pre-immunization antibodies against mtNUBC1 (dark purple), post-immunization antibodies against mtNUCB1-hIAPP (pink), and post-immunization antibodies against mtNUBC1 (light purple).*

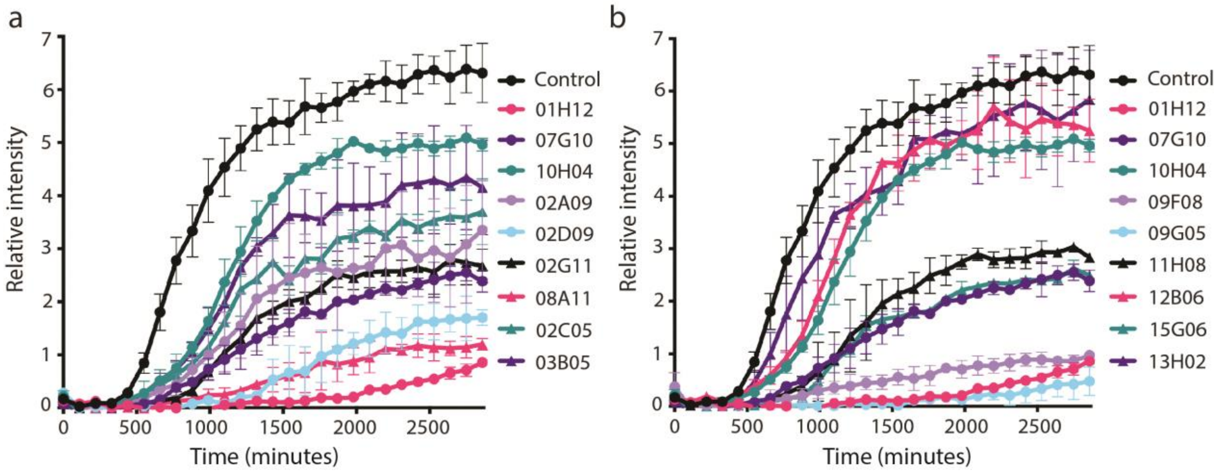

Figure S2. Kinetic analysis of Ab clones via ThT assay. *Kinetic analysis screening of various candidate mAbs (A, B) showing various degrees of aggregation inhibition. 07G10 (purple circle), and 10H04 (teal circle) were selected for further analysis. Clones were selected with ThT inhibition that suggested binding to small or intermediate protofibrils as opposed to monomeric or large fibrils. n = 2 per antibody. Error bars represent S.E.M.*

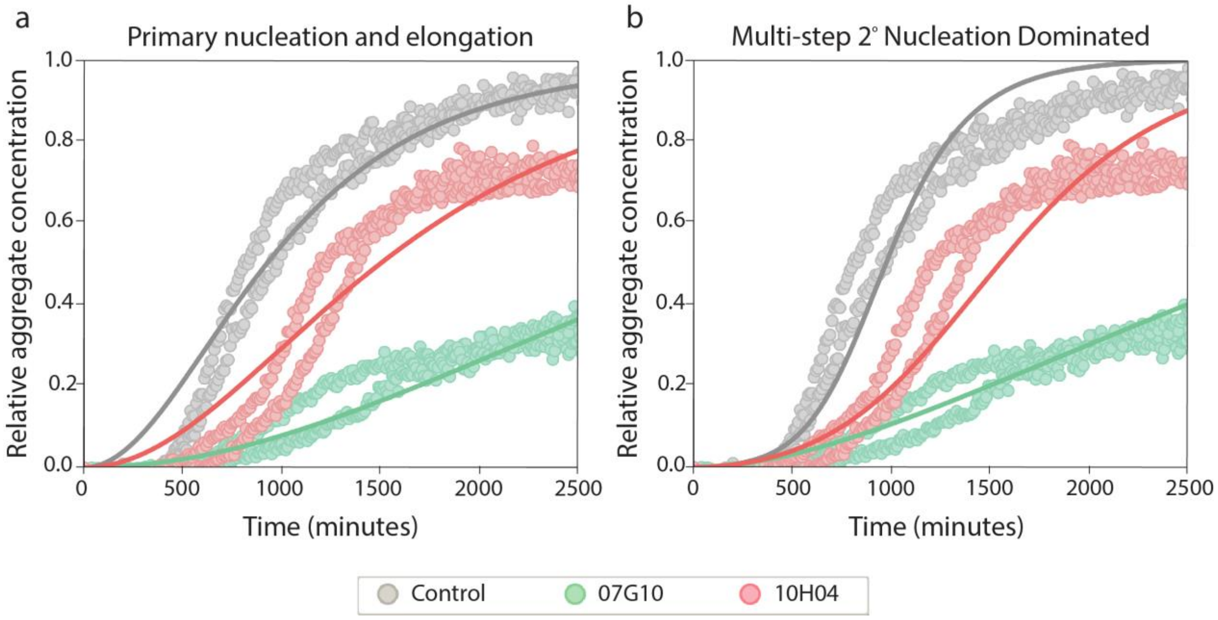

Figure S3. AmyloFit 2.0 analysis of control, 07G10, and 10H04. *AmyloFit 2.0 models for primary nucleation and elongation (A) and multi-step secondary nucleation dominated (B) for control (gray), 07G10 (green) and 10H04 (red). Trendlines show multi-step secondary nucleation dominated as the best global fit for the data. Kinetic constants are shown in Table*

S3. n=2 per antibody. Mean residual errors for A and B are 4.63e-03 and 6.9e-03, respectively.

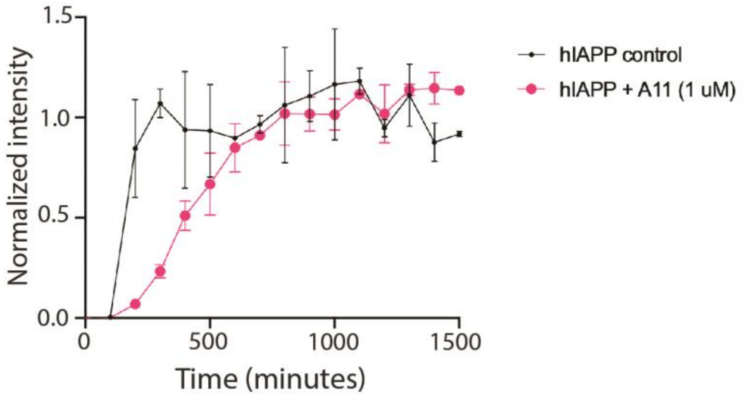

***Figure S4. A11 recognizes hIAPP protofibril mixture**. Kinetic analysis of A11 antibody (1 µM) via ThT assay showing aggregation inhibition supportive of A11 binding to pre-fibrillar stage of hIAPP (2.5 µM). n=2 per group. Error bars represent S.E.M.*

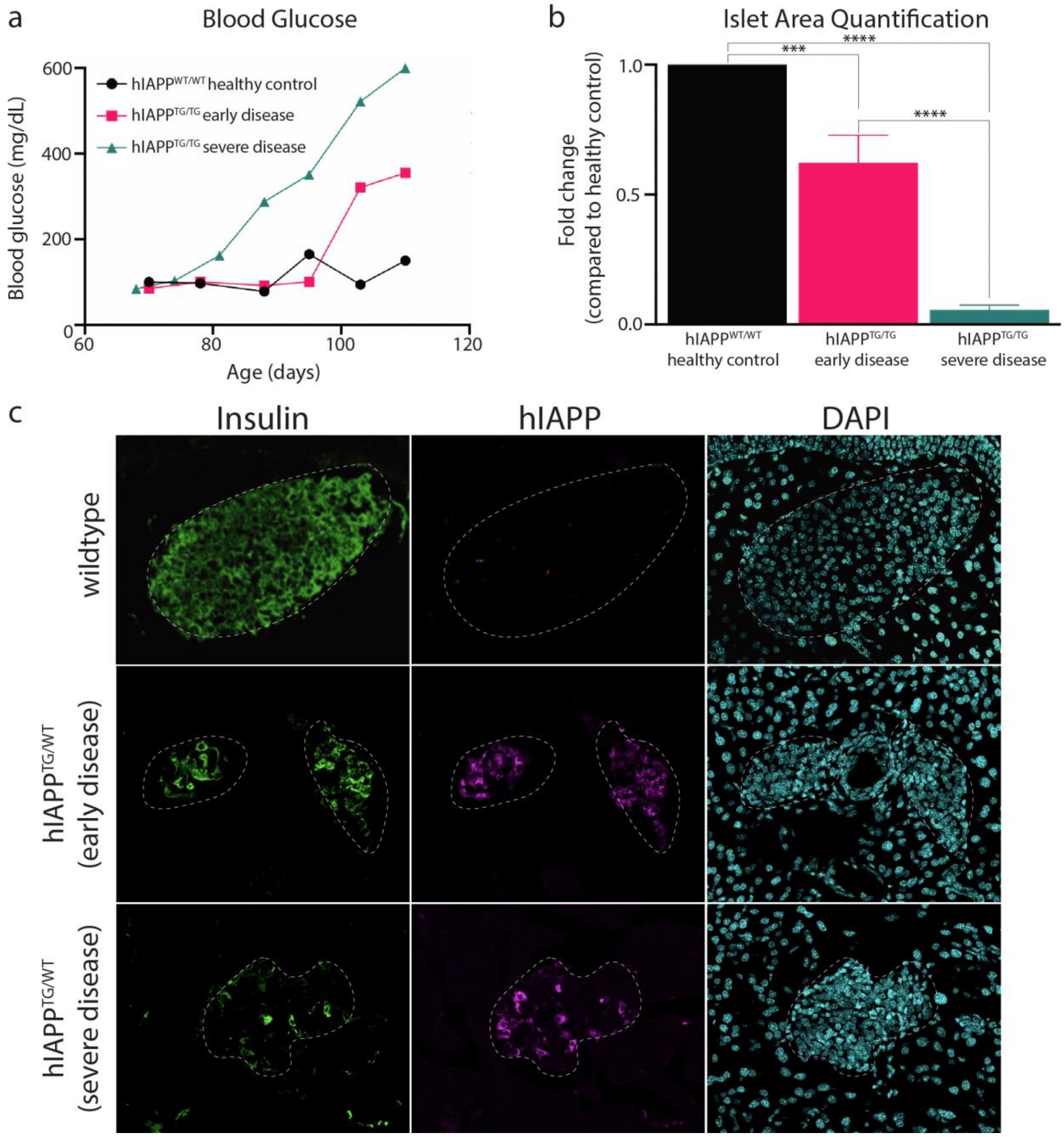

Figure S5. hIAPP$^{TG/TG}$ mice show increasing disease severity over time*. A) Fasting blood glucose levels of healthy WT, early diseased, and severely diseased transgenic mice. B) Quantification of insulin-positive islet area showing progressive disease. C) Immunofluorescence showing hIAPP expression, decreased insulin expression, and increased macrophage infiltration with disease progression. ****p<0.0001, ***p<0.001.*

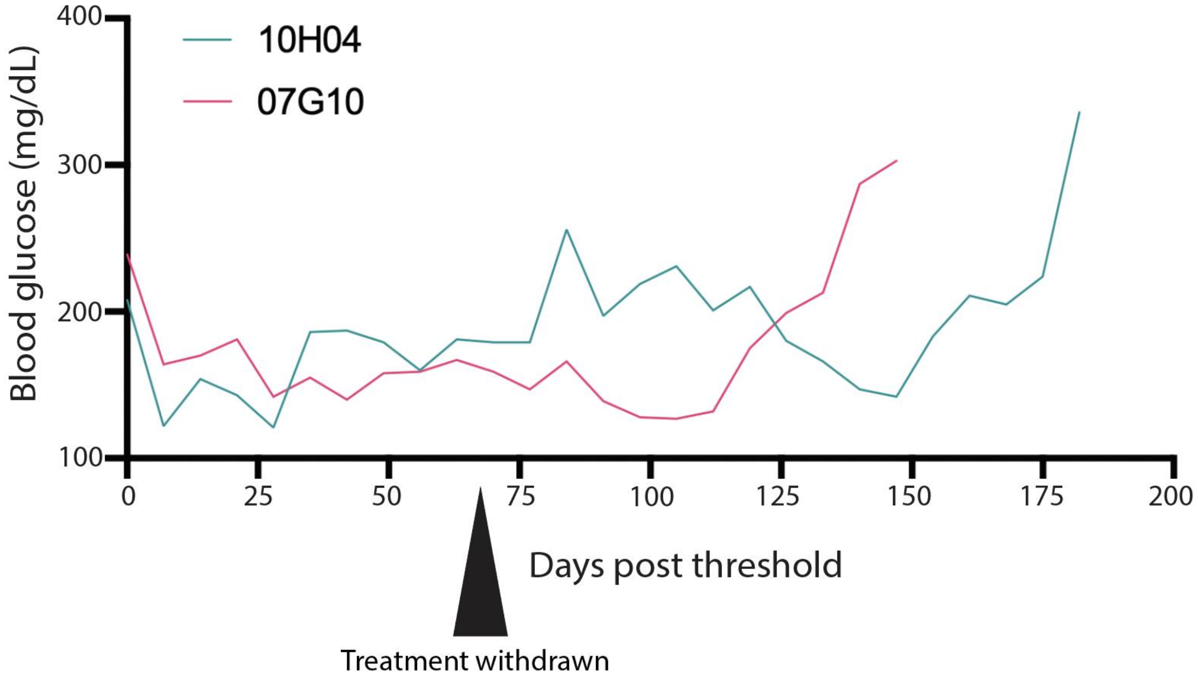

Figure S6. mAb treated mice show sustained response weeks after treatment withdrawal. *One mouse from each group that showed sustained response to treatment had treatment withdrawn after 10 weeks. Fasting blood sugars began rising steadily after 5 weeks without 07G10 treatment (magenta) and 10 weeks without 10H04 treatment.*

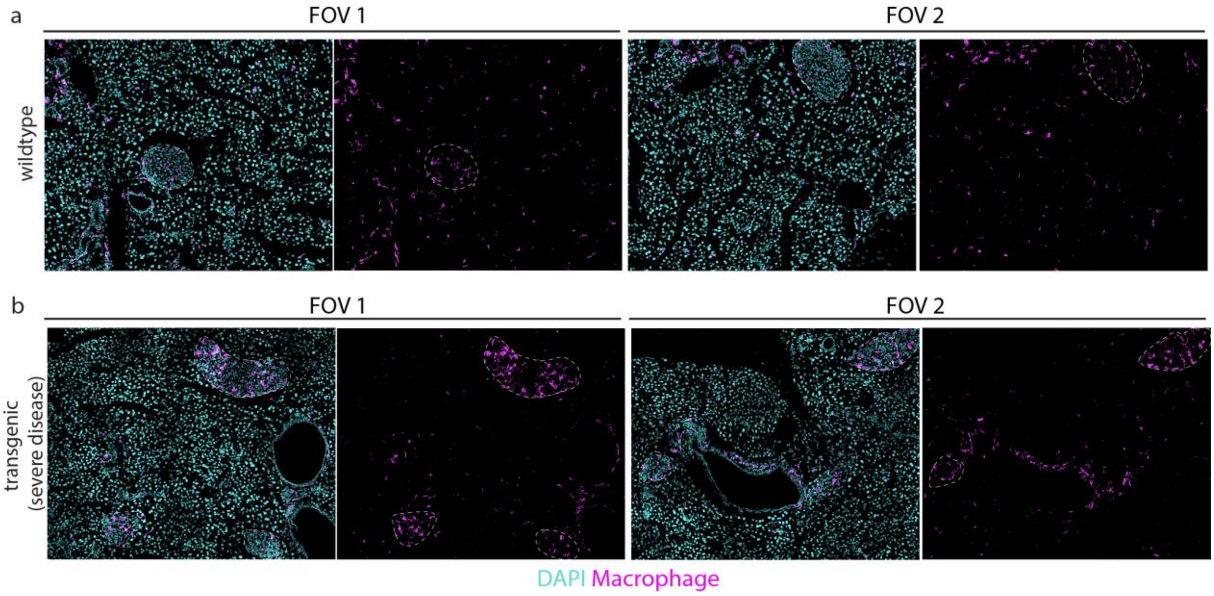

Figure S7. hIAPP$^{TG/TG}$ mice show macrophage homing to islets. *A) Stitched images from two fields of view (FOV) of pancreatic sections from hIAPP wildtype mice showing macrophage signal in the exocrine and endocrine pancreas (islets). B) Stitched images from two fields of view of pancreatic sections from diabetic hIAPP$^{TG/TG}$ showing a distinct enrichment/ infiltration of macrophages away from the exocrine pancreas into the endocrine pancreas. Islets are outlined in white dashed lines. Macrophage (magenta), nuclei (cyan).*

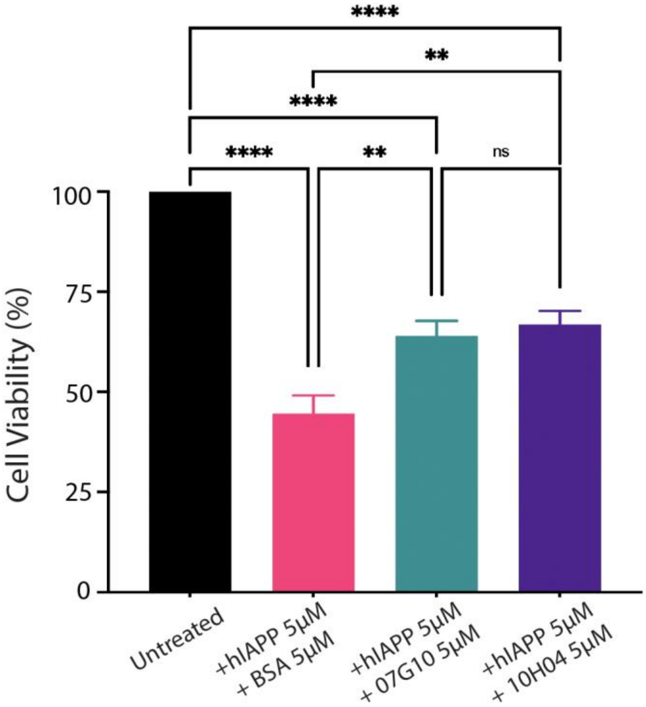

Figure S8. mAbs show protection against hIAPP cytotoxicity in vitro. *PrestoBlue cell assay showing cell viability 24 hours after the addition of 5 µM hIAPP with the addition of 5 µM BSA control, 5 µM 07G10 or 5 µM 10H04. Cell viability was normalized to untreated cells. n = 5 technical replicates for each condition. Error bars represent S.E.M, ordinary one-way ANOVA. ****p<0.0001, **p<0.01, ns=not significant.*

Table S1: Kinetic data of 2.5µM hIAPP aggregation were analyzed with AmyloFit 2.0. Initial monomer concentration (m_0), initial fibril number concentration (P_0), initial fibril mass concentration (M_0), reaction order of primary nucleation (n_c), and reaction order of secondary nucleation (n_2) parameters were set to Global constants. Each time one of the rate constants (primary nucleation, elongation, or secondary nucleation) was set to 'Fit' while the others were set to 'Global fit'. Each table represents a single fit where P_0, m_0, n_c, M_0 and n_2 were kept as global constants, and only one of the rate constants was individually fitted while the other two were globally fitted.

Fitting for Nucleation

Dataset<br>*Units*	P_0<br>µM	m_0<br>µM	n_c<br>unitless	k_n<br>$\mu M^{(-nc+1)} min^{-1}$	k_+<br>$\mu M^{-1} min^{-1}$	M_0<br>µM	k_2<br>μM^{n2}	n_2<br>unitless
hIAPP 2.5 µM + 1 µM BSA	0	2.5e-06	2	6.09	2.71e+04	0		
hIAPP 2.5 µM + 1 µM 07G10	0	2.5e-06	2	0.502	2.71e+04	0		
hIAPP 2.5 µM + 1 µM 10H04	0	2.5e-06	2	2.32	2.71e+04	0		

Fitting for Elongation

Dataset<br>*Units*	P_0<br>µM	m_0<br>µM	n_c<br>unitless	k_n<br>$\mu M^{(-nc+1)} min^{-1}$	k_+<br>$\mu M^{-1} min^{-1}$	M_0<br>µM	k_2<br>μM^{n2}	n_2<br>unitless
hIAPP 2.5 µM + 1 µM BSA	0	2.5e-06	2	1.56e-04	2.71e+04	0		
hIAPP 2.5 µM + 1 µM 07G10	0	2.5e-06	2	1.56e-04	2.71e+04	0		
hIAPP 2.5 µM + 1 µM 10H04	0	2.5e-06	2	1.56e-04	2.71e+04	0		

Fitting for Secondary Nucleation

Dataset<br>*Units*	P_0<br>µM	m_0<br>µM	n_c<br>unitless	k_n<br>$\mu M^{-1} min^{-1}$	k_+<br>$\mu M^{-1} min^{-1}$	M_0<br>µM	k_2<br>μM^{n2}	n_2<br>unitless
hIAPP 2.5 µM + 1 µM BSA	0	2.5e-06	2	21.8	1.11e+03	0	1.00e+09	2
hIAPP 2.5 µM + 1 µM 07G10	0	2.5e-06	2	21.8	1.11e+03	0	6.60e-03	2
hIAPP 2.5 µM + 1 µM 10H04	0	2.5e-06	2	21.8	1.11e+03	0	2.16e+08	2

Table S2. Human tissue sample data

Patient Sample	*Age*	*Health status*	*Tissue type*
T1234188	77	Normal (non-diabetic)	Pancreatic sections
T1236188Dia	68	Diabetes, Type II	Pancreatic sections
NBP2-77581	77	Normal (non-diabetic)	Pancreatic sections
NBP2-77739	68	Diabetes, Type II	Pancreatic sections

Table S3. hIAPP peptide information

Product	*Vendor*	*Lot #*	*Molecular Formula*	*Molecular Mass (Da)*	*I.D. (MALDI-MS)*	*Purity (HPLC)*
Amylin (human) trifluoroacetate salt	BACHEM	3016548	$C_{165}H_{261}N_{51}O_{55}S_2$	3909.33	3909.78	95.1%
H-Lys-Cys-Asn-Thr-Ala-Thr-Cys-Ala-Thr-Gln-Arg-Leu-Ala-Asn-Phe-Leu-Val-His-Ser-Ser-Asn-Asn-Phe-Gly-Ala-Ile-Leu-Ser-Ser-Thr-Asn-Val-Gly-Ser-Asn-Thr-Tyr-NH_2 trifluoroacetate salt (Disulfide bond)						

Table S4: hIAPP peptide amino acid analysis. Observed and estimated composition reported in numbers of residues. *Partially destroyed during acid hydrolysis. **Histidine value low due to the resistance of the Val-His bond to acid hydrolysis.

Amino Acid	*Observed Composition*	*Estimated Composition*
Asx	6.08	6
Ala	4.11	4
Tyr	0.88	1
Thr	5.52	5
*Cys**	0.04	2
Phe	2.04	2
Ser	4.57	5
Val	2.02	2
*His***	0.87	1
Glx	1.03	1
Ile	0.88	1
Lys	1.05	1
Gly	2.02	2
Leu	2.92	3
Arg	0.99	1

Two novel mAbs are reported that bind hIAPP protofibrils and inhibit their aggregation and toxicity to pancreatic islets in a mouse model of rapidly progressing type 2 diabetes mellitus (T2D). The mAbs can detect serum protofibrils in diabetic animals and mAb treatment causes impressive increases in survival. These data introduce the possibility of new diagnostic and therapeutic avenues in T2D.

A. S. Bortoletto, W. V. Graham, G. Trout, A. Bonito-Oliva, M. A. Kazmi, J. Gong, E. Weyburne, B. L. Houser, T. P. Sakmar, R. J. Parchem*

Human Islet Amyloid Polypeptide (hIAPP) Protofibril-Specific Antibodies for Detection and Treatment of Type 2 Diabetes

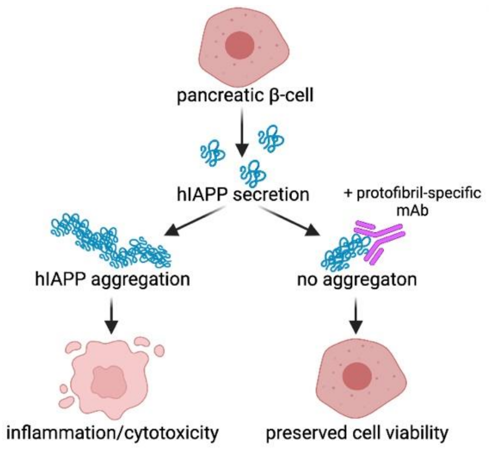

Supplement: Supplementary file 1 — Supporting Information [file ADVS-9-2202342-s001.pdf]
